# Responses of human adipose-derived mesenchymal stem cells to chemical microenvironment of the intervertebral disc

**DOI:** 10.1186/1479-5876-10-49

**Published:** 2012-03-16

**Authors:** Chengzhen Liang, Hao Li, Yiqing Tao, Xiaopeng Zhou, Fangcai Li, Gang Chen, Qixin Chen

**Affiliations:** 1 Department of Orthopedics, 2nd Affiliated Hospital, School of Medicine, Zhejiang University, No. 88 Jie fang Road, Hangzhou 310009, China

**Keywords:** Adipose-derived mesenchymal stem cells, Intervertebral disc, Chemical microenvironment

## Abstract

**Background:**

Human adipose-derived mesenchymal stem cells (ADMSCs) may be ideal source of cells for intervertebral disc (IVD) regeneration, but the harsh chemical microenvironment of IVD may significantly influence the biological and metabolic vitality of ADMSCs and impair their repair potential. This study aimed to investigate the viability, proliferation and the expression of main matrix proteins of ADMSCs in the chemical microenvironment of IVD under normal and degeneration conditions.

**Methods:**

ADMSCs were harvested from young (aged 8-12 years, n = 6) and mature (aged 33-42 years, n = 6) male donors and cultured under standard condition and IVD-like conditions (low glucose, acidity, high osmolarity, and combined conditions) for 2 weeks. Cell viability was measured by annexin V-FITC and PI staining and cell proliferation was measured by MTT assay. The expression of aggrecan and collagen-I was detected by real-time quantitative polymerase chain reaction and Western blot analysis.

**Results:**

IVD-like glucose condition slightly inhibited cell viability, but increased the expression of aggrecan. In contrast, IVD-like osmolarity, acidity and the combined conditions inhibited cell viability and proliferation and the expression of aggrecan and collagen-I. ADMSCs from young and mature donors exhibited similar responses to the chemical microenvironments of IVD.

**Conclusion:**

IVD-like low glucose is a positive factor but IVD-like high osmolarity and low pH are deleterious factors that affect the survival and biological behaviors of ADMSCs. These findings may promote the translational research of ADMSCs in IVD regeneration for the treatment of low back pain.

## Background

Low back pain is a public health problem, which continues to be a common disability that reduces the quality of life of the patients [1]. Low back pain is a multifactorial disease, and intervertebral disc (IVD) degeneration plays an important role in its etiology [2,3]. IVD degeneration involves the reduction of disc cells and the extracellular matrix, which consists predominantly of proteoglycans, collagens, and noncollagenous proteins [4]. Current treatments for the diseases resulting from IVD degeneration are mainly aimed at relieving symptoms of low back pain, which could not prevent the progression of IVD degeneration or restore disc structure and function [5]. Therefore, there is an urgent need to understand the pathogenesis of IVD degradation to develop effective therapies for low back pain.

Although disc cells constitute only 1% of the adult disc tissue by volume, they play a significant role in matrix synthesis and the maintenance of a healthy IVD tissue [6]. IVD degeneration is accompanied by a decrease in the number of disc cells, suggesting that cell transplantation is a potential biological therapy approach for IVD degeneration. Autologous disc cells may be an ideal cell source, but they have many practical limitations in the clinical setting: (1) The procurement of autologous disc cells, whether accomplished by image-guided aspiration or open surgical collection, is an invasive process; (2) The harvesting of disc cells from a healthy IVD can potentially accelerate IVD degeneration; (3) Disc cells from a degenerated disc may not be functionally ideal for re-implantation [7].

Adipose-derived mesenchymal stem cells (ADMSCs) emerge as a better candidate for cell therapy because of their ease access, little donor site morbidity and high proliferation rate [8-11]. These stem cells are able to differentiate into nucleus pulposus (NP)-like cells and secrete extracellular matrix consisting of anionic proteoglycans, collagen-II and aggrecan [12]. However, the chemical microenvironment of IVD is harsh, which is characterized by limited nutrition, high osmolarity, acidity and low oxygen tension [13]. NP cells have been shown to be well adapted to this harsh microenvironment [14], but IVD microenvironment may negatively influence the biological and metabolic vitality of ADMSCs and impair their repair potential.

Wuertz *et al*. investigated the behaviors of rat bone marrow mesenchymal stem cells (BM-MSCs) in IVD microenvironment and found that low glucose maintained cell proliferation and enhanced matrix biosynthesis whereas high osmolarity and low pH conditions were critical factors that reduced cell proliferation and matrix biosynthesis of BM-MSCs [15]. However, it remains unknown the response of ADMSCs to IVD microenvironment under normal and degenerated conditions. Therefore, the purpose of this study was to investigate the viability, proliferation and the expression of main matrix proteins of ADMSCs in the chemical microenvironment of IVD under normal and degeneration conditions.

## Materials and methods

### ADMSCs isolation and culture

**Figure 1:**
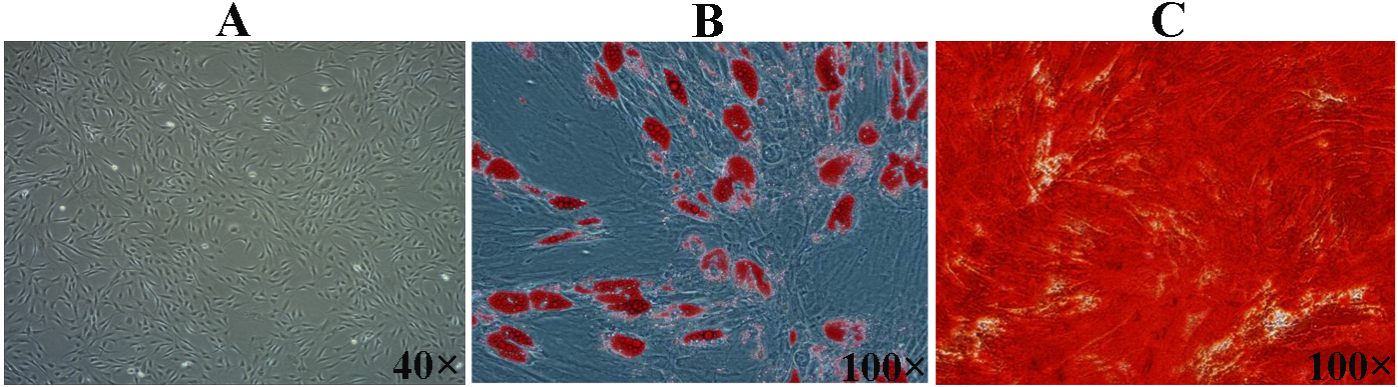
**Characterization of isolated human ADMSCs by osteogenic and adipogenic differentiation**. A: Human ADMSCs were expanded in monolayer culture (×40). B: ADMSCs were cultured in osteogenic media for 21 d and stained positive with alizarin red. Red staining marked mineral deposition in their newly formed extracellular matrix (×100). C: ADMSCs were cultured in adipogenic media for 14 d and stained positive with Oil red O staining. The lipid drop appeared red under fluorescence microscope (×100).

The isolation of ADMSCs was performed following the approval of Ethics Committee of Zhejiang University with informed consent of the patients. Human ADMSCs were isolated from subcutaneous adipose tissues obtained from young (aged 8-12 years, n = 6) and mature (aged 33-42 years, n = 6) male donors undergoing elective surgical procedures. Approximately 1.5 g of adipose tissues were washed with phosphate buffered saline (PBS) and finely minced, then were digested with 0.15% collagenase type I (Sigma, St. Louis, MO) at 37°C for 30 min in a water-bath shaker (200 rpm). The collagenase was inactivated by the addition of Dulbecco's Modified Eagle Medium (DMEM) supplemented with 10% fetal calf serum (FCS), Penicillin (50 units/ml), Streptoymcin (50 *μ*g/ml). The ADMSCs-containing cell suspension was centrifuged at 600 g for 5 min. The isolated cells were plated in 25 cm^2 ^cell culture flasks at a density of 2.5 × 10^4 ^cells and cultured in standard culture medium at 37°C with 5% CO2. Cultures were washed with PBS after 48 h to remove unattached cells, and re-fed with fresh medium. ADMSCs were expanded up to the passage 2 and were characterized by osteogenic and adipogenic differentiation. Osteogenic differentiation of ADMSCs using an osteogenesis kit (Cyagen Biosciences, Guangzhou, China) was confirmed by positive alizarin red (Sigma, St. Louis, MO) staining of mineralized matrix after 21 days of culture (Figure 1B). Adipogenic differentiation of ADMSCs using an adipogenesis kit (Cyagen Biosciences, Guangzhou, China) was confirmed by Oil Red O (Sigma) staining of lipid droplets after 14 days of culture (Figure 1C).

For cell culture, all reagents were purchased from Invitrogen (Carlsbad, CA, USA). Standard culture medium was Dulbecco's modified Eagle's medium (DMEM) supplemented with 10% fetal calf serum (FCS), Penicillin (50 units/ml), and Streptomycin (50 μg/ml). IVD-like glucose medium was prepared by using a low glucose DMEM medium (1.0 mg/mL). IVD-like osmolarity medium was prepared by adding a mixed solution of NaCl (5 M) and KCl (0.4 M), which increased the osmolarity of the medium from 280 to 485 mOsm [15]. For IVD-like acidic pH medium, the pH was adjusted to 6.8 using a commercial pH microelectrode (Lazarlab, Los Angeles, CA) by adding 1 M HCl. The IVD-like combined medium was prepared as follows: the pH was 6.8, osmolarity was 485 mOsm, and glucose content was 1.0 mg/mL (Table 1). The IVD-like pH and combined media were incubated at 37°C with 5% CO2 for 3 days in order to confirm that the pH was stable.

**Table 1:** The prepared culture media

	DMEM (4.5 mg/mL)	DMEM (1.0 mg/mL)	FCS (%)	Penicillin (U/mL)	Streptoymcin (μg/mL)	5 M NaCl 0.4 M KCl (%)	pH	characteristics
Standard medium	√		10	50	50		7.4	4.5 mg/mL glucose 280 mOsm pH 7.4

IVD-like glucose medium		√	10	50	50		7.4	1.0 mg/mL glucose 280 mOsm pH 7.4

IVD-like osmolarity medium	√		10	50	50	2	7.4	4.5 mg/mL glucose 485 mOsm pH 7.4

IVD-like pH medium	√		10	50	50		6.8	4.5 mg/mL glucose 280 mOsm pH 6.8

IVD-like combined medium		√	10	50	50	2	6.8	1.0 mg/mL glucose 485 mOsm pH 6.8

ADMSCs of passage 2 were seeded either in 24-well plates for cell proliferation assay or in 25 cm^2 ^cell culture flasks for cell viability and gene expression analysis. After 24 h, culture media were changed to either standard media or specific media. ADMSCs were cultured in specific media for 2 weeks with media changes every 48 h.

### Flow cyotomtry analysis

Viability of cells was determined by annexin V-FITC and PI (AV-PI) staining as described previously [16]. Viable cells were defined as those negative for annexin V-FITC and PI staining, apoptotic cells were defined as those positive for annexin V-FITC and negative for PI staining, and secondary necrotic cells were defined as those positive for annexin V-FITC and positive for PI staining. After 2 weeks of culture, the cells were harvested and incubated with FITC-labeled annexin V and PI for 30 min in the dark at 4°C. Then the cells were analyzed by FACS with a FACS Calibur cytometer (FACScan, Becton Dickinson) equipped with CellQuest software (BD Biosciences, San Jose, CA).

### MTT assay

Cell proliferation was assessed by MTT (3-[4, 5-dimethylthiazol-2-yl]-2, 5-diphenyltetrazolium bromide) assay. Briefly, cells were plated at a density of 1.5 × 10^4 ^cells in 24-well plates and cultured for 1 week or 2 weeks. Then the medium was replaced with 500 μl of MTT at 5 mg/ml (0.25 mg MTT in DMEM, Sigma) and incubation was continued for 4 h at 37°C. Finally, the formazan crystals were solubilized with 200 μl sterile dimethyl sulfoxide (DMSO) (Sigma) and absorbance was measured at 570 nm by using a Spectra MAX microplate reader (Molecular Devices, Sunnyvale, California, USA).

### Real-time quantitative polymerase chain reaction (RT-PCR)

Total RNA was isolated from the cells using Trizol (Sigma) according to the manufacturer's instructions. Subsequently, 2 μg of total RNA was reverse transcribed using 1 μl of oligo-dT_18 _primer (Invitrogen, Carlsbad, CA, UK), 25 units of RNase inhibitor, 1 μl of dNTPs (10 mM), and 200 U of reverse transcriptase (Superscript II, Gibco/BRL). The quantification of mRNA expression levels for aggrecan and collagen-I were carried out on an iCycler system (Bio-Rad, Laboratories, Hercules, CA, USA). iQ™SYBR Green super mix PCR kit (Bio-Rad) was used for real-time monitoring of amplification (5 ng of template cDNA; 45 cycles: 95°C/10 s, 62°C/25 s) with appropriate primers (Table 2). Human 18 s rRNA was used as internal control. The cycle threshold (CT) value for each gene was corrected using the mean CT value. RT-PCR data were quantified using the ΔCT method with the formula: n = 100 × 2^- (ΔCT targeted gene-ΔCT GAPDH)^. The primers used were 18 s rRNA forward: 5'-GACTCAACACGGGAAACCTCAC-3' and reverse: 5'-CCAGACAAATCGCTCCACCAAC-3' (122 bp); Aggrecan, forward: 5'- GGCATTTCAGCGGTTCCTTCTCCA -3' and reverse: 5'- ACGATCCACTCCTCCACACCAGA -3' (81 bp); Collagen I, forward: 5'-GGTGTTGTGCGATGACGTGATCTGT-3' and reverse: 5'-CTTGGTCGGTGGGTGACTCTG-3' (112 bp).

### Western blot analysis

Cells were washed with iced PBS and then lysed in ice-cold lysis buffer (Cell Signaling Technology) by using a rotor/stator homogenizer (2,000 rpm, three times for 10 s each). The protein contents of the lysates were analyzed by BCA method and equal amount of 60 μg protein was separated by 10% SDS-PAGE. The separated proteins were transferred to polyvinylidene difluoride (PVDF) membrane. After being blocked with 5% non-fat milk in Tris-buffered saline containing 0.05% Tween 20 (TBST), the membrane was incubated with following primary antibodies: monoclonal rabbit monoclonal rabbit anti-aggrecan (1:1000; Santa Cruz), anti-collagen-I (1:8000; Santa Cruz) and monoclonal rabbit anti-β-actin (1:1000; Santa Cruz) in TBST overnight at 4°C. The membrane was then washed three times with TBST and incubated with horseradish peroxidase-coupled secondary antibody (1:1000; Santa Cruz) in TBST for 1 h at room temperature. The immunoreactive protein was detected using ECL (GE Healthcare) and BioMaxfilm (Kodak). The relative amount of immunoreactive protein in each sample was quantitated by densitometry (AMBIS Radioanalytic and Visual Imaging System, Ambis, USA).

### Statistical analysis

ADMSCs cultured under standard condition served as a control group. A two way ANOVA with Post-hoc comparisons was used to analyze the differences between experiment and control groups, followed by Fisher's PLSD. Statistical analyses were performed with SPSS 17.0 for Windows. Results were expressed as mean ± standard deviation (SD). Statistical significance was set at *P *< 0.05.

## Results

### Effects of chemical microenvironments on the viability of ADMSCs

**Figure 2:**
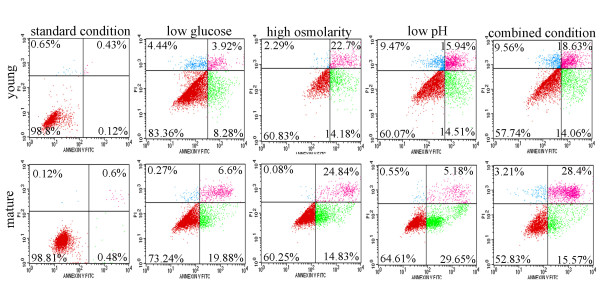
**The viability of ADMSCs under IVD-like chemical conditions**. The numbers of viable, apoptotic, and secondary necrotic cells were determined by annexin V-FITC and PI (AV-PI) staining. Compared to the standard condition, IVD-like glucose condition slightly decreased the number of viable cells while IVD-like osmolarity, IVD-like pH and combined conditions significant decreased the number of viable cells and increased the number of apoptotic cells.

**Figure 3:**
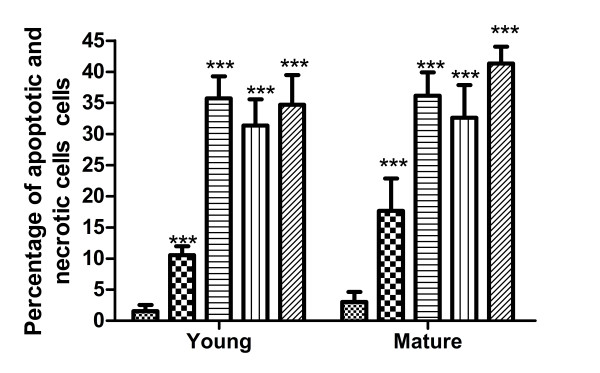
**Percentage of apoptotic and necrotic cells of ADMSCs under IVD-like chemical conditions**. Flow cytometry analysis showed a slight increase in the number of apoptotic cells under IVD-like low glucose condition, a decrease in the number of vital cells and an increase in the number of apoptotic and necrotic cells under IVD-like osmolarity, IVD-like pH as well as combined IVD-like conditions. Data were presented as mean ± SD (n = 6). Statistically significant differences were indicated with asterisks, *** *P *< 0.001.

By flow cytometry analysis we observed a slight increase in the number of apoptotic cells under IVD-like low glucose condition, but a decrease in the number of vital cells and an increase in the number of apoptotic and necrotic cells under IVD-like osmolarity, IVD-like pH as well as combined IVD-like conditions (Figure 2 and 3). ADMSCs harvested from young and mature donors had similar cell viability under the same condition.

### Effects of chemical microenvironments on the proliferation of ADMSCs

**Figure 4:**
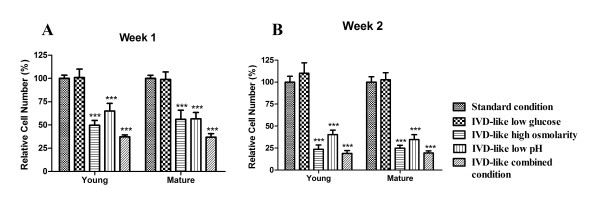
**The proliferation of ADMSCs under IVD-like chemical conditions**. MTT assay of ADMSCs cultured for 1 week or 2 weeks under IVD-like chemical conditions. Data were presented as mean ± SD with *P *< 0.05 for n = 6 (young males) and n = 6 (mature males). Statistically significant differences were indicated with asterisks, *** *P *< 0.001.

Effects of chemical microenvironments on cell proliferation were examined after 1 week or 2 weeks of culture (Figure 4). The proliferation of ADMSCs from both young and mature donors was significantly inhibited by IVD-like microenvironments after 1 week and 2 weeks of culture (both *P *< 0.001, 2-way ANOVA).

ADMSCs harvested from young and mature donors exhibited a similar response to the microenvironments of IVD, although cells from young donors exhibited slightly higher proliferation rate. Under low glucose condition, the proliferation of both young and mature MSCs was similar to that under the standard condition after 1 week and 2 weeks. Under high osmolarity and low pH conditions, the proliferation of both age groups was strongly inhibited after 1 week (49.63% young, 55.94% mature under osmolarity condition, both *P *< 0.001; 65.09% young, 56.63% mature under pH condition, both *P *< 0.001) and 2 weeks (23.45% young, 24.85% mature under osmolarity condition, both *P *< 0.0001; 40.11% young, 34.66% mature under pH condition, both *P *< 0.001). Under combined conditions, cell proliferation was also strongly inhibited after 1 week (37.25% young, 36.89% mature, both *P *< 0.001) and 2 weeks (49.63% young, 55.94% mature, both *P *< 0.001).

### Effects of chemical microenvironments on the expression of aggrecan and collagen-I in ADMSCs

**Figure 5:**
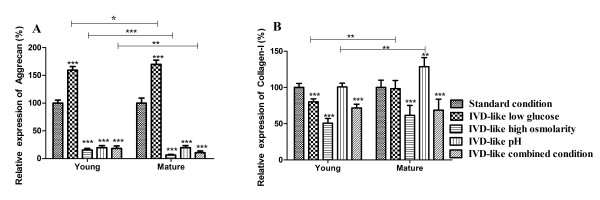
**mRNA expression of aggrecan and collagen-I in ADMSCs under the IVD-like chemical conditions**. RT-PCR analysis of aggrecan and collagen-I mRNA levels in ADMSCs cultured for 1 week or 2 weeks under IVD-like chemical conditions. Statistically significant differences were indicated with asterisks, * *P *< 0.05, ** *P *< 0.01, *** *P *< 0.001.

After 2 weeks of exposure to IVD-like conditions, the mRNA expression of aggrecan and collagen-I in ADMSCs was detected by RT-PCR (Figure 5). The results showed a significant effect of chemical conditions (*P *< 0.001) and age (*P *< 0.001) as well as a trend for an interaction between chemical conditions and age (*P *< 0.001) on aggrecan and collagen-I expression. IVD-like glucose condition increased aggrecan mRNA expression in both groups (159.48% young, 170.00% mature, both *P *< 0.001) and slightly decreased collagen-I mRNA expression in young group (79.95%, *P *< 0.001), but had no effect on collagen-I mRNA expression in mature group. IVD-like osmolarity significantly decreased mRNA expression of aggrecan (15.31% young, 6.52% mature, both *P *< 0.001) and collagen-I (50.71% young, 61.28% mature, both *P *< 0.001) in both groups. IVD-like pH significantly decreased aggrecan mRNA expression (19.74% young, 19.68% mature, both *P *< 0.001) and increased collagen-I mRNA expression in mature group (128.59%, *P *= 0.005), but had no effect on collagen-I mRNA expression in young group. IVD-like combined conditions inhibited mRNA expression of aggrecan (18.17% young, 10.83% mature, both *P *< 0.001) and collagen-I (71.62% young, 68.73% mature, both *P *< 0.001).

There were age differences for aggrecan mRNA expression under low glucose (*P *= 0.027), high osmolarity (*P *< 0.001), and combined conditions (*P *= 0.009). The significant age differences were also observed for collagen-I mRNA expression under low glucose (*P *= 0.004) and low pH conditions (*P *= 0.001).

**Figure 6:**
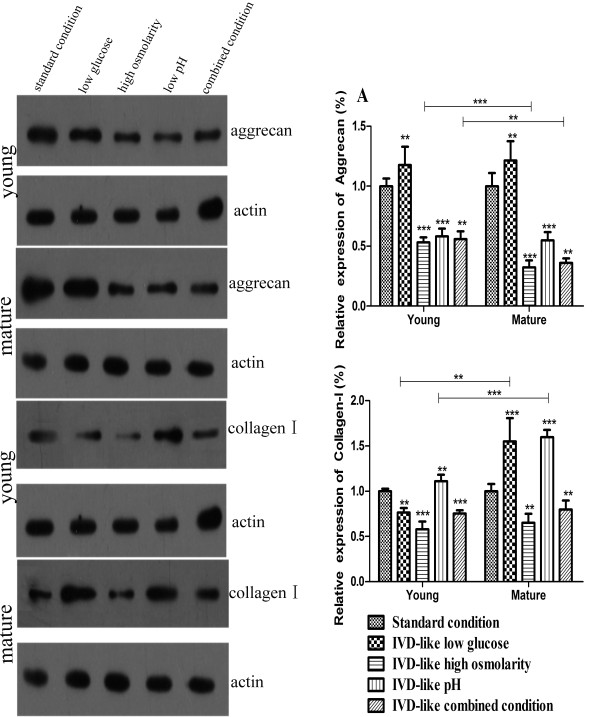
**Protein expression of aggrecan and collagen-I in ADMSCs under the IVD-like chemical conditions**. Western blot analysis of aggrecan and collagen-I protein levels in ADMSCs cultured for 1 week or 2 weeks under IVD-like chemical conditions. Shown were representative blots from six independent experiments with similar results. *β*-actin served as loading control. Statistically significant differences were indicated with asterisks, ** *P *< 0.01, *** *P *< 0.001.

In addition, the expression of aggrecan and collagen-I at protein level in ADMSCs cultured for 2 weeks was detected by Western blot analysis (Figure 6). The results showed significant effects of chemical conditions (*P *< 0.001) and age (*P *< 0.001) as well as an interaction between chemical conditions and age (*P *< 0.001) on aggrecan and collagen-I protein expression. IVD-like glucose condition slightly increased aggrecan protein expression (117.71% young, *P *= 0.002; 121.49% mature, *P *= 0.001) in both groups and collagen-I expression in mature age group (164.33% mature, *P *= 0.005), but decreased collagen-I expression in young group (76.36% young, *P *< 0.001). IVD-like osmolarity significantly decreased aggrecan protein expression (53.13% young, 32.18% mature, both *P *< 0.001) and collagen-I protein expression (57.95% young, *P *< 0.001; 65.06% mature, *P *= 0.001). IVD-like pH significantly decreased aggrecan protein expression (58.18% young, 54.82% mature, both *P *< 0.001), but increased collagen-I expression (111.02% young, *P *= 0.003; 159.70% mature, *P *< 0.001) in both groups. IVD-like combined conditions significantly inhibited the expression of aggrecan (55.76% young, 35.96% mature, both *P *< 0.001) and collagen-I (75.15% young, *P *< 0.001; 79.73% mature, *P *= 0.035) in both groups.

There were significant age differences for aggrecan protein expression under high osmolarity (*P *< 0.001) and combined conditions (*P *= 0.009). The significant age differences were also observed for collagen-I protein expression under low glucose (*P *= 0.001) and low pH conditions (*P *< 0.001). These results were almost consistent with the findings of RT-PCR analysis.

## Discussion

One of the primary causes of low back pain is IVD degeneration, which is characterized by a decrease in cell density and a reduction in the synthesis of cartilage-specific extracellular matrix components [17,18]. Recent studies have focused on the use of cell-based tissue engineering to regenerate IVD and several of them demonstrated that ADMSCs are capable of differentiating into chondrocyte-like cells of the NP *in vitro *[19] and *in vivo *[6,20], suggesting the potential of ADMSCs-based therapies to restore functional IVD in degenerated discs. However, the biological and metabolic vitality of ADMSCs may be influenced by the harsh microenvironment of IVD characterized by limited nutrition, high osmolarity, acidity and low oxygen tension. Few studies have attempted to examine the response of ADMSCs to the chemical microenvironment of the IVD under normal and degenerative conditions. With this in mind, here we designed a study to investigate the viability, proliferation and expression of matrix proteins of ADMSCs harvested from young and mature donors under a variety of conditions that represented the chemical microenvironment of IVD.

The IVD is the largest avascular tissue in human body with distances of up to 8 mm from the center of an adult lumbar disc to the nearest blood supply [21]. Disc cells are supplied with essential nutrients by the diffusion from the blood supply through mainly the cartilaginous endplates and disc tissue [22-24]. During IVD degeneration, loss of endplate permeability will result in a decrease in the nutrient supply [25]. It is proposed that reduced nutrient levels could be inadequate to maintain cellular viability or activity, leading to IVD degeneration [26-28]. In the present study we found that the reduction in glucose supply slightly reduced cell viability, consistent with previous studies about the influence of low glucose on the viability of NP cells [27,29]. In addition, in agreement with an earlier study on matrix synthesis of BM-MSCs under low glucose condition [15], we found that the expression of aggrecan was significantly up-regulated in both age groups and the expression of collagen-I was not significantly influenced. Based on these data we conclude that IVD-like low glucose in the *in vivo *conditions may be a positive factor for ADMSCs transplantation, because it maintained cell viability and proliferation at relatively normal levels and enhanced the expression of matrix proteins.

Aggrecan, the major type of proteoglycan, is present on the matrix of annulus under physiological condition and is negatively charged [30]. These negative charges account for the high osmotic pressure of the IVD. Loading of the IVD leads to the changes in its hydration and osmolarity. It is reported that osmotic pressure in the NP varies from 450 mOsm to 550 mOsm [13]. The changes in osmolarity could affect the signaling cascades in the cells. Mavrogonatou *et al*. found that increased osmolarity activated p38 MAPK and triggered the ATM-p53-p21^WAF1^-pRb pathway in disc cells, which arrested the cells in the G2 and G1 phase of the cell cycle [31]. Although low glucose condition may be the limiting nutrient for the survival of disc cells, we found that high osmolarity appeared to be more critical to ADMSCs. Our results show that IVD-like high osmolarity significantly reduced the viability and proliferation of ADMSCs. High osmolarity also strongly inhibited the expression of aggrecan and collagen-I, which was not consistent with a previous study reporting that hyperosmolarity had no significant effects on proteoglycan content but up-regulated collagen-I expression in NP and anulus fibrosus (AF) cells [32]. The precise mechanisms are unknown but the differences in cell adaptability may account for the inconsistent results. The concentration of proteoglycans is positively correlated with the osmolality of extracellular matrix in IVD and the loss of proteoglycans leads to a decrease of osmotic pressure during IVD degeneration [33]. Therefore, we conclude that IVD-like high osmolarity may be a critical factor for ADMSCs transplantation.

The acidic pH is proposed to be detrimental to the activity of disc cells because the rates of proteoglycan synthesis in disc explants have been shown to decrease markedly *in vitro *at low pH level [34-36]. Our results show that IVD-like pH caused a significant decrease in cell viability, cell proliferation and aggrecan expression of ADMSCs, providing further evidence that pH is a critical factor limiting the biological and metabolic vitality of ADMSCs. It was noteworthy that expression of collagen-I was not changed or slightly increased under IVD-like pH, although the acidic pH caused a significant decrease in cell viability and proliferation. This observation was consistent with the expression of collagen-I in ADMSCs when transplanted into the degenerative IVD characterized by low pH. Ganey *et al*. reported that the expression of collagen-I in the degenerative IVD was not significantly different between the dogs received ADMSCs transplantation and no intervention controls [6]. The precise mechanism is not known, and further studies are necessary to provide the explanation.

Finally, we observed that the combined conditions had the most significant effect on the biological and metabolic vitality of ADMSCs. Because low glucose maintained cell viability, cell proliferation and biosynthesis at relatively normal levels, we speculate that osmolarity and acidity are significant microenvironmental factors that affect the survival and biological behaviors of ADMSCs.

Previous studies have reported that MSCs from old donors exhibited a decreased maximal life span and decreased potential of proliferation and differentiation, compared to MSCs from young donors [34-36]. However, our study showed only minor age effects with regards to the viability and proliferation of ADMSCs under chemical conditions of IVD. Therefore, further studies are necessary to investigate whether ADMSCs harvested from young or mature donors is better to be utilized for IVD regeneration.

There are a number of limitations for the present study. First, the donor pool of ADMSCs was heterogeneous, which may contribute to the observed variations in cell viability, proliferation, and gene and protein expression. Second, the choice for 2D culture system allowed the pH level to be controlled easily compared to the 3D culture. However, this simplification would disrupt cell-matrix interactions and cellular signal transduction. In addition, collagen-II expression was not detectable, consistent with the findings that monolayer culture is known to decrease collagen-II expression and increase collagen-I expression [37]. Third, the regulation of matrix turnover is a complex process and involves many proteins. In this study we only examined the expression of representative markers of matrix biosynthesis and degradation. Future studies should include more donors and examine whether the expression of other matrix proteins, such as biglycan, decorin and lumican will change under the chemical microenvironment of IVD. In addition, further investigation based on 3D culture system will be essential as an important complement to the standard cell culture system used in this study.

## Conclusion

IVD-like low glucose is a positive factor for ADMSCs-based IVD regeneration by maintaining cell viability and proliferation at relatively normal levels and enhancing matrix biosynthesis of ADMSCs. In contrast, IVD-like high osmolarity and low pH are deleterious factors that inhibit cell viability and proliferation and matrix biosynthesis. These findings may promote the translational research of ADMSCs in IVD regeneration for the treatment of low back pain.

## Competing interests

The authors declare that they have no competing interests.

## Authors' contributions

CZL and HL performed research, analyzed data and wrote the paper; YQT, XPZ and GC analyzed data; YQT, XPZ and FCL performed research; QXC designed research and wrote the paper. The authors reported no potential conflicts. All authors read and approved the final manuscript.
